# Two Independent Positive Feedbacks and Bistability in the Bcl-2 Apoptotic Switch

**DOI:** 10.1371/journal.pone.0001469

**Published:** 2008-01-23

**Authors:** Jun Cui, Chun Chen, Haizhu Lu, Tingzhe Sun, Pingping Shen

**Affiliations:** State Key Laboratory of Pharmaceutical Biotechnology, School of Life Sciences, Nanjing University, Nanjing, People's Republic of China; RIKEN Genomic Sciences Center, Japan

## Abstract

**Background:**

The complex interplay between B-cell lymphoma 2 (Bcl-2) family proteins constitutes a crucial checkpoint in apoptosis. Its detailed molecular mechanism remains controversial. Our former modeling studies have selected the ‘Direct Activation Model’ as a better explanation for experimental observations. In this paper, we continue to extend this model by adding interactions according to updating experimental findings.

**Methodology/Principal Findings:**

Through mathematical simulation we found bistability, a kind of switch, can arise from a positive (double negative) feedback in the Bcl-2 interaction network established by anti-apoptotic group of Bcl-2 family proteins. Moreover, Bax/Bak auto-activation as an independent positive feedback can enforce the bistability, and make it more robust to parameter variations. By ensemble stochastic modeling, we also elucidated how intrinsic noise can change ultrasensitive switches into gradual responses. Our modeling result agrees well with recent experimental data where bimodal Bax activation distributions in cell population were found.

**Conclusions/Significance:**

Along with the growing experimental evidences, our studies successfully elucidate the switch mechanism embedded in the Bcl-2 interaction network and provide insights into pharmacological manipulation of Bcl-2 apoptotic switch as further cancer therapies.

## Introduction

Apoptosis is a highly regulated cell suicide program in response to cell stress, damage or conflicting division signals [1], [2]. Once decision is made, the demise of cell is all-or-none, irreversible [3], [4]. Mitochondria play a central role in apoptosis through sensing incoming cytotoxic signals and responding by mitochondrial outer membrane permeabilization (MOMP). MOMP is considered as the ‘point of no return’ of mitochondria-dependent cell death [5]. Once MOMP has occurred, pro-apoptogenic factors such as cytochrome c, Smac/DIABLO, and Omi/Htra 2, are released from intermembrane space (IMS) into cytosol where they initiate multiple cell death pathways irreversibly [6].

It has been widely embraced that the intricate interplay between three groups of the Bcl-2 family proteins determines the commitment of MOMP and subsequent apoptosis [5]. The group of BH-3-only proteins (include Bim, Bad, Bid, Bik, Bmf, Puma, Noxa and Hrk) serve as upstream sentinels that selectively respond to specific, proximal death signals by increasing expressions and/or post-translational regulations. Somehow they activate the group of multidomain proteins (include Bax and Bak), which are considered the critical mediators of apoptosis. Both in vivo and in vitro experiments proved that activated Bax/Bak can effectively cause MOMP by forming oligomerized pores (MAC, mitochondrial apoptosis channel) on mitochondrial outer membrane (MOM) [7]. Another anti-apoptotic group (include Bcl-2, Bcl-xL, Bcl-W, Mcl-1 and A1) has the opposite effect despite that they share a similar three-dimensional structure with the multidomain group.

**Figure 1:**
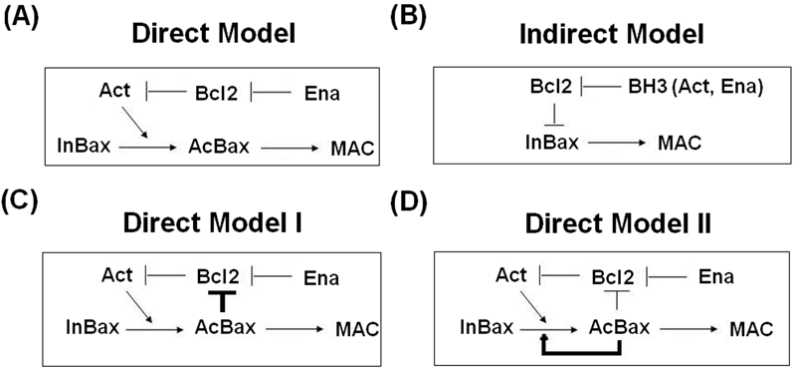
Schematic representation of the models of the Bcl-2 apoptotic switch. (A) The Direct Model. (B) The Indirect Model. (C) The Direct Model I, considering Activated Bax/Bak-anti-apoptotics interaction. (D) The Direct Model II, considering Bax/Bak auto-activation. Abbreviations used: Ena (Enabler), Act (Activator), Bcl2 (Anti-apoptotics), InBax (No-activated Bax/Bak), AcBax (Activated Bax/Bak), MAC (Bax oligomer, Mitochondrial Apoptosis Channel), BH-3 (BH-3 only proteins). Arrows represent material flow and catalyzed reaction. Black lines terminated by a bar denote interaction and inhibition.

Although growing evidence demonstrates Bcl-2 family functions as an upstream life/death switch [8], [9], detailed molecular mechanism of the so-called ‘Bcl-2 apoptotic switch’ remains controversial. Whether Bax/Bak is activated directly or indirectly is a central question. A widely accepted model proposes that the select BH-3-only proteins termed ‘activators’(tBid, Bim and Puma) is sufficient to enable a conformational change and pore formation of Bax/Bak in a ‘hit and run’ manner (Fig. 1A). Other BH-3-only proteins termed ‘enabler’ (also called de-repressor or sensitizer) can displace the activators from anti-apoptotic proteins [10]. Recently Kim and his coworkers provided compelling evidence to support this Direct Activation Model [11]. Another scenario supported by Willis et al. is that anti-apoptotic proteins inhibit apoptosis by sequestering Bax/Bak (Fig. 1B) [12], [13]. All BH-3-only proteins activate Bax/Bak indirectly by binding and inactivating their specific anti-apoptotic relatives. Nevertheless, this Indirect Activation Model is based on a hypothesis that Bax/Bak alone can spontaneously undergo conformational change and oligomerize without external activation steps, which still lacks experimental confirmation. Also, our previous modeling studies suggest that the Direct Activation Model is more plausible to explain ultrasensitive responses as well as other salient features of the Bcl-2 apoptotic switch in contrast to the Indirect Activation Model [14].

Now, quickly accumulated experimental evidence in this field can help update our understandings for the regulation of Bcl-2 network. As reviewed by Galonek and Hardwick [10], it is evident that anti-apoptotics can bind directly with activated form of Bax/Bak, however the interaction between anti-apoptotics and non-activated Bax/Bak remains suspectable. This fact criticized the Indirect Activation Model in which non-activated Bax/Bak-anti-apoptotics interaction is essential. In addition, Dlugosz et al. proved that Bcl-2, one of the major anti-apoptotics integrated in MOM, have to be ‘activated’ as well before inhibiting Bax oligomerization [15]. Later, Peng et al. suggested that this conformational change of Bcl-2 can only be induced by either tBid (one of the BH-3 only proteins), or activated Bax upon interaction, but not non-activated Bax [16]. Their findings further emphasized the importance of activated Bax-anti-apoptotics interaction. Another issue concerns Bax/Bak auto-activation. Tan et al. provided solid evidence that activated Bax can directly induce a conformational change in non-activated Bax, and anti-apoptotics can prevent Bax auto-activation through binding activated Bax [17]. Similar observations have also been reported in Bak [18]. Obviously, these new interactions can significantly influence the dynamical pattern of the models we discussed before.

In this study, we extended the Direct Activation Model by incorporating these newly emerged experimental findings. We mainly focused on their influence on the dynamic patterns in the extending models. Our analysis demonstrates that two independent positive feedback loops in the Bcl-2 network contribute to an inherent switch to govern apoptosis decision. Bistability can arise from a positive (double negative) feedback provided by the interaction between anti-apoptotics and activated Bax/Bak. Moreover, Bax/Bak auto-activation as another positive feedback can enforce this switch and make it more robust with respect to parameter variations. We also use ensemble stochastic modeling to elucidate the influence of intrinsic noise on this bistable switch. Our result is well in accord with certain experimental data of flow-cytometry analysis, where bimodal Bax activation distributions in cell population are reported. In all, our studies successfully extend our understanding of the switch mechanism beyond the apoptosis decision and provide insights into pharmacological manipulation of Bcl-2 family members as further cancer therapies.

## Materials and Methods

### Model Description

We constructed our models of Bcl-2 apoptotic switch mainly based on the previously described Direct Model (Fig. 1A) [14]. To reduce the complexity of the network without sacrificing fundamental components of the network, we group Bcl-2 family members into four distinct categories: Bax, Bcl2, Activator, and Enabler, representing multidomain pro-apoptotic member, anti-apoptotic members, Activators, and Enablers respectively. Two newly discovered mechanisms are incorporated by extending our models. Thus we obtain 3 models with increasing mechanistic details for further simulation and analysis (Fig. 1A, C and D). Many pro-death signals initiate apoptosis by regulating the expression of both anti- and pro-apoptotic Bcl-2 proteins [19]. Pro-apoptotic signals can also control apoptosis by affecting degradation rate of apoptosis related proteins, such as Bax [20]. So in this paper we considered the production and degradation rate of diverse Bcl-2 family members instead of cytotoxic stress as model inputs. In addition, increasing evidence supports that activated Bax/Bak is the major component of the cytochrome c release channel MAC [21]. Therefore, in our simulations the percentage of activated Bax/Bak is used to evaluate the degree of apoptosis.

### Computational modeling

Here we bring forward a set of models realizing mass action kinetics implemented as ODEs, which is based on the outline of the Direct Model, Direct Model I and Direct Model II as shown in Fig. 1. For each model, the state of a cell is described by the concentrations of all relevant molecules (c_1_, c_2_ … c_n_). The reaction rates are dependent on these concentrations and on biochemical parameters (k_1_, k_2_ … k_m_) such as binding constants and dissociation constants. To describe the temporal behavior, a set of ordinary differential equations is generated in terms of the following general equation:
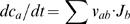
where *dc_a_/dt* represents the concentration changing rate of molecule *a*. *J_b_* denotes the rate of reaction *b*, and *v_ab_* denotes the stoichiometric matrix linking the reaction rates of *J_b_* with the affected molecule *a*. Here the reaction scheme, kinetic equations as well as model parameters of the Direct Model II are represented in Table 1, Table 2 and Table 3, respectively. And we can get the Direct Model and the Direct Model I by setting related reaction rates to 0 based on the Direct Model II (Direct Model: *J_2_ = J_4_ = J_8_ = J_9_ = J_AcBaxBcl2_ = 0,* see Table S1 and S2 in the supplementary material; Direct Model I: *J_9_* = 0, see Table S3 and S4 in the supplementary material). ODE23s routine was used for deterministic modeling, while explicit tau-leap method was used for stochastic modeling (The Mathworks, Natick, MA). The bifurcation analysis was done in XPPAUT (Version 5.91).

**Table 1:** Chemical reaction network scheme of the Direct Model II.^a^

No.	Reactions	Description	k+	k−
1	InBax+Act→AcBax+Act	Act-mediated InBax activation	k1	-
2	AcBax+Bcl2↔AcBaxBcl2	AcBax-Bcl2 dimerization and dissociation	k2	k3
3	Act+Bcl2↔ActBcl2	Act-Bcl2 dimerization and dissociation	k4	k5
4	AcBax+ActBcl2↔AcBaxBcl2+Act	Displacement between AcBax and Act	k6	k7
5	AcBax→InBax	AcBax inactivation	k8	-
6	Ena+Bcl2↔EnaBcl2	Ena-Bcl2 dimerization and dissociation	k9	k10
7	Act+EnaBcl2↔ActBcl2+Ena	Displacement between Act and Ena	k12	k11
8	AcBax+EnaBcl2↔AcBaxBcl2+Ena	Displacement between AcBax and Ena	k14	k13
9	InBax+AcBax→MAC	Bax auto-activation and dimerization	k15	-
10	2AcBax↔MAC	AcBax dimerization and dissociation	k16	k17
11	InBax↔Φ	InBax degradation and production	p1	u1
12	AcBax→Φ	AcBax degradation	-	u2
13	Act↔Φ	Act degradation and production	p2	u3
14	Bcl2↔Φ	Bcl2 degradation and production	p3	u4
15	ActBcl2→Φ	ActBcl2 degradation	-	u5
16	AcBaxBcl2→Φ	AcBaxBcl2 degradation	-	u6
17	Ena↔Φ	Ena degradation and production	p4	u7
18	EnaBcl2→Φ	EnaBcl2 degradation	-	u8
19	MAC→Φ	MAC degradation	-	u9
				

a Abbreviations used: InBax (Inactive Bax/Bak), Act (Activator), AcBax (Activated Bax/Bak), Bcl2 (Anti-apoptotics), AcBaxBcl2 (Activated Bax/Bak-Bcl2 dimer), ActBcl2 (Activator-Bcl2 dimer), Ena (Enabler), EnaBcl2 (Enabler-Bcl2 dimer), MAC (Bax/Bak pore), Φ (null).

**Table 2:** Ordinary differential equations (ODEs) of the Direct Model II.

*d*[*InBax*]/*dt* = *J_InBax_*−*J*_1_−*J*_9_	
*d*[*AcBax*]/*dt* = *J_AcBax_*+*J*_1_−*J*_2_−*J*_4_−*J*_5_+*J*_8_−*J*_9_−2·*J*_10_	
*d*[*Bcl*2]/*dt* = *J_Bcl_*_2_−*J*_2_−*J*_3_−*J*_6_	
*d*[*Act*]/*dt* = *J_Act_*−*J*_1_−*J*_3_+*J*_4_+*J*_7_	
*d*[*ActBcl*2]/*dt* = *J_ActBcl_*_2_+*J*_3_−*J*_4_−*J*_7_	
*d*[*AcBaxBc*l2]/*dt* = *J_AcBaxBcl_*_2_+*J*_2_+*J*_4_−*J*_8_	
*d*[*Ena*]/*dt* = *J_Ena_*−*J*_6_−*J*_7_−*J*_8_	
*d*[*EnaBcl*2]/*dt* = *J_EnaBcl_*_2_+*J*_6_+*J*_7_+*J*_8_	
*d*[*MAC*]/*dt* = *J_MAC_*+*J*_9_+*J*_10_	
*With*	
*J_InBax_* = *p*_1_−*u*_1_·[*InBax*]	*J*_1_ = *k*_1_·[*InBax*]·[*Act*]
*J_AcBax_* = −*u*_2_[*AcBax*]	*J*_2_ = *k*_2_·[*AcBax*]·[*Bcl*2]−*k*_3_·[*AcBaxBcl*2]
*J_Act_* = *p*_2_−*u*_3_·[*Act*]	*J*_3_ = *k*_4_·[*Act*]·[*Bcl*2]−*k*_5_·[*ActBcl*2]
*J_Bcl_*_2_ = *p*_3_−*u*_4_·[*Bcl*2]	*J*_4_ = *k*_6_·[*AcBax*]·[*ActBcl*2]−*k*_7_·[*AcBaxBcl*2]·[*Act*]
*J_ActBcl_*_2_ = −*u*_5_·[*ActBcl*2]	*J*_5_ = *k*_8_·[*AcBax*]
*J_AcBaxBcl_*_2_ = −*u*_6_·[*AcBaxBcl*2]	*J*_6_ = *k*_9_·[*Ena*]·[*Bcl*2]−*k*_10_·[*EnaBcl*2]
*J_Ena_* = *p*_4_−*u*_7_·[*Ena*]	*J*_7_ = *k*_11_·[*Ena*]·[*ActBcl*2]−*k*_12_·[*Act*]·[*EnaBcl*2]
*J_EnaBcl_*_2_ = −*u*_8_·[*EnaBcl*2]	*J*_8_ = *k*_13_·[*Ena*]·[*AcBaxBcl*2]−*k*_14_·[*AcBax*]·[*EnaBcl*2]
*J_MAC_* = −*u*_9_·[*MAC*]	*J*_9_ = *k*_15_·[*InBax*]·[*AcBax*]
	*J*_10_ = *k*_16_·[*AcBax*]^2^−*k*_17_·[*MAC*]

**Table 3:** Parameters of the Direct Model II.^a^

Parameters	Description	Value^b^	Ref. and comment
[InBax]_0_	Initial concentration of InBax	60	Same as in [14]
[Act]_0_	Initial concentration of Act	1	Same as in [14]
[Bcl2]_0_	Initial concentration of Bcl2	30	Same as in [14]
[Ena]_0_	Initial concentration of Ena	1	Similar as in [14]
p1	Production rate of InBax	0.06	Estimated from [14], [33]
p2	Production rate of Act	0.001	Estimated from [14], [33]
p3	Production rate of Bcl2	0.03	Estimated from [14], [33]
p4	Production rate of Ena	0.001	Estimated from [14], [33]
u1	Degradation rate of InBax	0.001	Similar as in [33]
u2	Degradation rate of AcBax	0.001	Same as u1
u3	Degradation rate of Act	0.001	Same as u1
u4	Degradation rate of Bcl2	0.001	Same as u1
u5	Degradation rate of Act-Bcl2 dimer	0.005	Similar as in [33]
u6	Degradation rate of Bax-Bcl2 dimer	0.005	Same as u5
u7	Degradation rate of Ena	0.001	Same as u1
u8	Degradation rate of Ena-Bcl2 dimer	0.005	Same as u5
u9	Degradation rate of Bax oligomer (MAC)	0.0005	Estimated
k1	Act-mediated Activation of Bax	0.0005	Similar as in [14], [33]
k2	Dimerization between AcBax and Bcl2	0.005	Similar as in [23]
k3	Dissociation of Bax-Bcl2 dimer	0.001	Same as in [14]
k4	Dimerization between Act and Bcl2	0.001	Similar as in [14], [23]
k5	Dissociation of Act-Bcl2 dimer	0.001	Same as k3
k6	AcBax displace Act from Act-Bcl2 dimer	0.005	Same as k2
k7	Act displace AcBax from Bax-Bcl2 dimer	0.001	Same as k4
k8	Bax/Bak inactivation	0.001	Same as in [14]
k9	Dimerization between Ena and Bcl2	0.0001	Similar as in [14], [23]
k10	Dissociation of Ena-Bcl2 dimer	0.001	Same as k3
k11	Ena displace Act from Act-Bcl2 dimer	0.0001	Same as k9
k12	Act displace Ena from Ena-Bcl2 dimer	0.001	Same as k4
k13	Ena displace AcBax from Bax-Bcl2 dimer	0.0001	Same as k9
k14	AcBax displace Ena from Ena-Bcl2 dimer	0.005	Same as k2
k15	Bax Auto-activation & dimerization	0.0002	estimated from k1
k16	Homo-dimerization of AcBax	0.0002	Same as k15
k17	Dissociation of Bax homo-dimer	0.02	Similar as in [40]

a To get the original Direct Model, k2, k6, k14, k15 are set to be zero. In the Direct Model I, k15 is set to be zero.

b Units: The total amounts of different species are expressed in units of nanomolar. The first and second rate constants are expressed in units of second^−1^ and nanomolar^−1^second^−1^, respectively. The production rate constants are expressed in unit of nanomolar/s.

### Parametric robustness analysis of bistability

Similar to [22], all parameters are varied +/−20% from its default value to generate 2000 random parameter sets by using Latin Hypercube Sampling. Since we still know little about the distributions of parameter values for real biological systems, we used uniform probability distribution for each parameter in this paper. For all the *in silico* simulations, the percentage of existence of bistability was monitored. The generation of Latin hypercubes and the determination of bistability are implemented in Matlab. For each set of kinetic parameters, we search for the existence of a bistability by the “coming up and going down” method described previously [23].

## Results

### Anti-apoptotics Establish an Positive Feedback Which Brings Bistability

A Bax-activation module has been identified as a bistable switch in our previous work without considering protein production and degradation [23]. However, protein production and degradation have been reported of great importance in apoptosis regulation [19], [20]. Here we continue to investigate whether bistability can originate from interactions of Bcl-2 family proteins in a model including protein synthesis and degradation. We first use the production rate of Activator as the apoptotic stimuli. Our results suggest that anti-apoptotics can establish a positive (double negative) feedback and bring bistability to the model.

**Figure 2:**
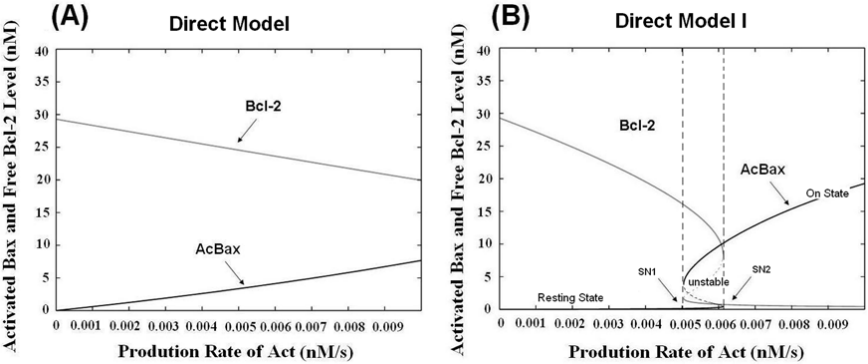
Steady states of Activated Bax/Bak (AcBax) and Anti-apoptotics (Bcl2) as a function of the production rate of Activator (Act). (A) The Direct Model. (B) The Direct Model I. The bistable region is enclosed by 2 vertical dashed lines.

We presented two models of increasing complexity to demonstrate how bistable behaviors of Bax activation emerge from the intricate interactions of Bcl-2 family proteins. From Fig. 2A we can see that no bistable behavior emerges from the Direct Model (Fig. 1A). However, Direct Model I (Fig. 1C), in which Bcl2 can also bind to activated Bax/Bak (AcBax), shows desired bistable behavior of Bax activation (Fig. 2B). This model exhibits three steady states, two stable (solid lines) and one unstable (dashed lines). The two saddle-node bifurcation points SN1 (0.0050) and SN2 (0.0061) enclose a bistable region (enclosed by 2 vertical dashed lines). Starting from the resting state, the system retains low level of Bax activation for increasing stimuli, until a threshold is reached (SN2), whereby the level of activated Bax/Bak switches (AcBax) to the higher stable state in an all-or-none fashion. The system remains at this higher state even if the stimulus is removed (between SN1 and SN2). Only when the stimulus passes another threshold (SN1) can the system return to its resting state. The level of monomer Bcl2 shows the opposite pattern as AcBax. This is a typical toggle-switch governed by the production rate of Activator. Parameters including the production and degradation rates of other proteins can also give bifurcation diagrams. For instance, bistability has been found when we use degradation rate of AcBax as the apoptotic stimuli (Supplementary materials, Fig. S1). Here for simplicity, we just used the production rate of Activator to represent the apoptotic stimuli, and show the bistability originated from interactions of Bcl-2 family proteins.

We next focused on the mechanism that leads to bistability of Bax activation. It is apparent that the inhibition of Bcl2 brings about ‘inhibitor ultrasensitivity’. That means the adding Activator is first inhibited till their amount exceed the inhibition effect of Bcl2. Also, we suggested that the interactions between AcBax and Bcl2 can form a positive (double negative) feedback. AcBax can bind Bcl2, and thereby sequesters Bcl2 away from the Activator. Thus more Bax can be activated. It is well known that positive feedback incorporated with non-linear elements can lead to bistability [24]. Here we can see that Bcl2, the anti-apoptotics play a pivotal role in eliciting bistable behavior in the Bcl-2 network.

### Bax Auto-activation as a Positive Feedback Enforces the Bistability

**Figure 3:**
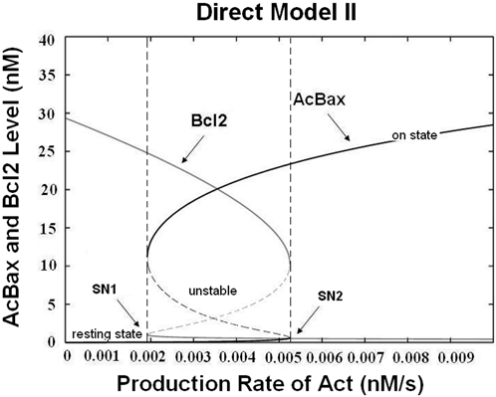
Bifurcation diagram of the Direct Model II. Steady states of Activated Bax/Bak (AcBax) and Anti-apoptotics (Bcl2) are plotted as a function of the production rate of Activator (Act). The bistable region is enclosed by 2 vertical dashed lines.

Direct Model II (Fig. 1D) which is a more detailed network involving Bax auto-activation also shows bistability (Fig. 3). It displays lower threshold of Bax activation and larger bistable domain than Direct Model I, which indicates that Bax auto-activation establishes a strong positive feedback loop which effectively enhances the bistable behavior. We can see that the bistable region is extended, and shifted from [0.0050, 0.0061] to [0.0019, 0.0052], compared to Direct Model I (Fig. 2B). The level of ‘on state’ of AcBax is also lifted higher in contrast to Direct Model I.

**Figure 4:**
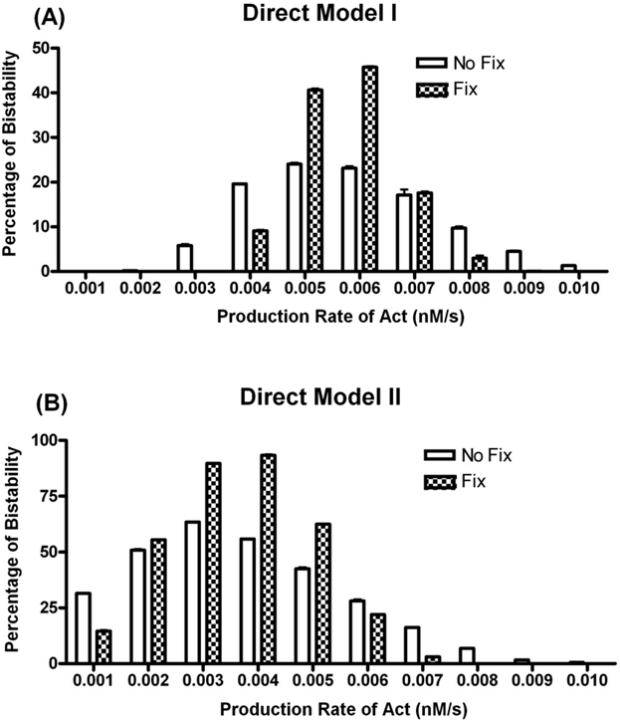
Parametric robustness of bistability. (A) The Direct Model I. (B) The Direct Model II. For any given production rate of Activator (0.001∼0.010), the percentage of parameter sets that exhibits bistability are plotted. No Fix: All of the parameters are varied +/−20% from its default value. Fix: The production rates of Bcl-2 family proteins (p1∼p4) are fixed while all other parameters are varied +/−20% from its default value for each sample run.

Bax auto-activation can also enforce model's robustness of the bistability. Here, we used a Monte Carlo based method to evaluate the robustness of bistable behavior of the Bcl-2 network to parameter variations. Latin Hypercube Sampling is used to generate 2000 random sets of all the parameters of the model with the variation of 40% (+/−20%) relative to the reference parameter values. From Fig. 4 (A and B, no Fix), we can see that above 20% of the total 2000 parameter sets for each models exhibit a bistable behavior in bistable regions ( [0.0050, 0.0061] of Direct Model I and [0.0019, 0.0052] of Direct Model II). These results indicate that the interactions of the Bcl-2 network can generate a good switch to control cell death in a certain window of parameter variations. In addition, when Bax auto-activation is involved (Direct Model II), the highest percentage of bistability is almost twice as the one in Direct Model I. The bistable range in respect to production rate of Activator is also broadened and moves to the left side of X-axis, which indicates that this positive feedback loop could significantly enhance the robustness of model system as well as the sensitivity in the face of apoptotic stimuli. In addition, we found a remarkable degree of robustness when the production rates of Bcl-2 family members are fixed in our model (Fig. 4, Fix). In the expected bistable region, higher percentages of bistability of both Models are reached. It indicates that the regulation of Bcl-2 family proteins' productions may play a key role in determining the bistable behavior of the system.

From the bifurcation analysis of these models, we demonstrate the potential molecular mechanism of the bistability arising from hierarchic interactions of the Bcl-2 network. A positive (double negative) feedback formed by activated Bax-Bcl-2 interactions could be an internal reason of the emergence of this bio-switch. Another possible positive feedback loop describing Bax auto-activation can enforce such behavior and make the bistable range wider and more robust with respect to parameter variations.

### Influences of the Intrinsic Noise on the Bcl-2 Apoptotic Switch

Most bistable models only maintain their switch-like behavior in a limited parameter range. These systems can hardly be considered ‘biologically bistable’ as they may not behave in a bistable manner in the face of biological uncertainties, such as intrinsic noise and parameter variations [25], [26]. We have examined the influence of parameter variations on the Bcl-2 apoptotic switch and have proved Bax auto-activation can improve the model's robustness of the bistability with respect to parameter variations. Here, we continue to investigate the effect of noise on the bistable Bcl-2 apoptotic switch by performing stochastic modeling of the Direct Model II. Actually, the effect of noise on bistable behaviors has already been discussed elsewhere [26]. Our major focus in this paper is not the general effect of noise on bistability but the mechanisms of bistability embedded in the Bcl-2 network. And as a specific example showing the influences of intrinsic noise on bistable systems, we successfully modeled how intrinsic noise can change ultrasensitive Bax activation switches into gradual responses in cell populations and how bimodal Bax activation distributions in cell populations are generated.

**Figure 5:**
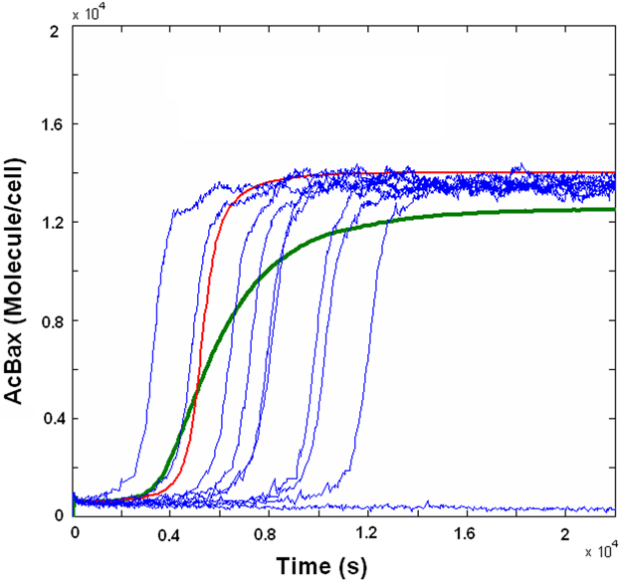
Time-series of the Activated Bax/Bak (AcBax) by deterministic and stochastic simulations. The production rate of Activator is set to be p_2_ = 0.0052 nM/s (estimated as 3.14 Molecule/s per cell). The initial concentration of Act is adopted as [Act]_0_ = 8 nM (estimated as 4800 Molecule per cell). The red line is the result from a deterministic simulation and the green line is the average of 10000 stochastic simulations. 10 of the stochastic simulation time-series are plotted as blue lines.

The time course of Bax activation in response to stimuli is simulated and the results indicate that the Bax activation switch is a robust bistable system since noise-induced transitions are rare unless the stimulus is very near the bifurcation point (Supplementary Materials, Fig. S2). Eissing et al. presented a similar result in their robustness analysis of two apoptosis models [27]. Here, we also investigated the effects of noise on the dynamic behaviors of Bax activation. Fig. 5 shows examples of stochastic simulations with an initial activator number of 8 nM (estimated as 4800 Molecule per cell, here estimating a cell volume of 1 picoliter results that 1 nM≈600 molecules per cell) and a production rate of Activator p2 = 3.14 Molecule/s per cell (estimated as 0.0052 nM/s). Red curve demonstrates the time course of Bax activation by deterministic model and blue curves are stochastic results. Both red and blue curves are ‘S-shape’, showing prominent switch-like behavior. Interestingly, intrinsic noise seems mainly to lead different time-delays of the transition from low activated state to high activated state of Bax compared to ‘standard form’ (red curve). Thus the result of averaging the sample paths (dashed curve) is no longer ‘S-shape like’ but gradual. Most experimental studies on Bax/Bak activation and oligomerization have been performed using cell populations (e.g. Western Blotting) where graded but not all-or-none behaviors are often observed [28], [29]. Those simulations give a plausible explanation that how the ‘all-or-none’ bistable behavior of single cell converts to observed gradual behavior of population data under noisy conditions.

**Figure 6:**
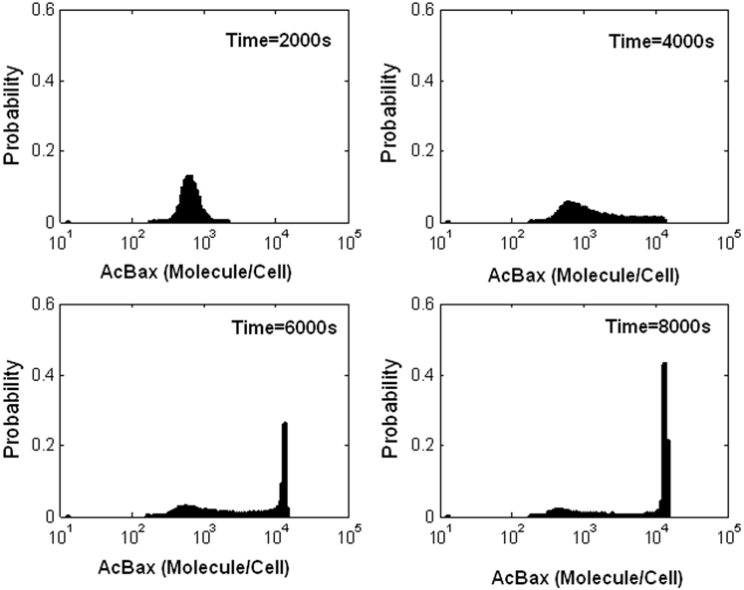
Probability distributions of Activated Bax/Bak (AcBax) molecule per cell as a function of time. Histograms are generated from the stochastic simulations at times 2000, 4000, 6000, 8000s of 10000 independent runs.

Now experimental data based on single cell monitoring techniques to confirm the Bax-activation switch proposed in our models are still lacking. One alternative is to examine whether a population of cells undergoing apoptosis can exhibit bimodal population distributions in Bax activation. Recently, a series of flow-cytometry experiments have detected a bifurcation of Bax activation into two states [30], [31]. Similar results have also been found in Bak activation [30]–[32]. These findings provide solid evidence to support our hypothesis that there exists a Bax/Bak-activation switch. The two independent positive feedbacks embedded in the Bcl-2 interaction network can provide a plausible explanation for this switch. Here we used ensemble modeling to prove how bimodal distribution can be generated from the switch under intrinsic noise. The sample paths (totally 10000 independent simulations) are used to produce histograms of the activated Bax at several different times (Fig. 6). Our modeling results show the desired transient binary response of Bax activation, which is consistent with these experimental observations.

## Discussion

Previously, most models explaining bistable behavior of apoptosis regard events downstream of mitochondria, such as caspases activation, as necessary parts in bistability [33]–[35]. However growing evidence has supported the importance of a so called ‘Bcl-2 apoptotic switch’ upstream of mitochondria [9]. The evidence include: (1) MOMP itself is all-or-none, irreversible [5], (2) MOMP can conduct apoptosis even its downstream events such as caspase activation are inhibited [36], (3) MOMP is mainly regulated by Bcl-2 family proteins [7], and (4) unrestrained Bax/Bak activation can act as the decision point to apoptosis [37]. Nevertheless, the molecular mechanism of the Bcl-2 apoptotic switch remains in hot debate. Multiple models can be cataloged into two distinct ideas, namely the Direct Activation Model and the Indirect Activation Model [10], [13]. Despite the contradicting experimental evidences, mathematical modeling provides a useful strategy to study the dynamical property and plausibility of these models.

In our former work we have identified a Direct Activation Model as a more plausible explanation for the switch-like behavior as well as three other salient features [14]. Also, in another paper we proposed a bistable model of Bax-activation by using both deterministic and stochastic modeling [23]. However it is still worthwhile to study whether bistability can originate in a model embracing emerging experimental findings as well as protein synthesis and degradation, which have been reported of great importance in apoptosis regulation (but not considered in our former modeling studies). In this paper we included updating knowledge into our former selected Direct Activation Model for the Bcl-2 apoptotic switch. Our modeling results successfully reveal that two positive feedbacks embedded in the Bcl-2 network can lead bistability and give a plausible explanation for the Bcl-2 apoptotic switch. We also showed that this switch can lead bimodal Bax-activation distribution of cell population by stochastic modeling, which is well in accord with recent experimental findings. Our analysis provides solid evidence that the Direct Activation Model is a plausible explanation for both Bax activation switch and apoptosis decision.

Although we believe Bcl-2 family proteins constitute a most important checkpoint upon mitochondria, it should be noted that various bistable switches downstream or independent of mitochondria have also been elucidated [33]–[35]. It would be quite interesting to study the cooperation of these switches when more numerical data is attainable to construct models of complete apoptosis network. Also, comparing these mechanisms can lead to interesting findings. It is surprising to find that the mechanism of bistability in the Bcl-2 apoptotic switch is quite similar to that described by Legewie et al. for a caspase activation switch [34]. In their model, activated Caspase3 can bind XIAP, and thereby sequesters XIAP away from activated Caspase9, which in turn activate more Caspase3. Quite similar to Bcl-2, XIAP establish a positive (double negative) feedback which brings bistability. In addition, there is also an independent positive feedback (Caspase3-mediated feedback cleavage of Caspase9) which enforces the bistability. Thus, we can conclude that there should be a general strategy adopted by cells in generating signaling switches: A weak non-liner positive feedback which generates bistability combined with a strong positive feedback which enforces the bistability.

Another conclusion is that the Direct Activation Model is more plausible than the Indirect Activation Model we describe before. Growing experimental evidence not only criticizes the basis for the Indirect Activation Model, but also proves that the Direct Activation Model can show bistability, a kind of switch. Also, we revealed that the bistability of the Direct Model is robust enough to withstand parameter variations as well as intrinsic noise. In contrast, the Indirect Model lacks both inhibition insensitivity ultrasensitivity and positive feedbacks which may contribute to switch-like responses.

The imbalance of Bcl-2 family protein expressions can cause a variety of diseases (e.g. cancers), which further proves the crucial therapeutic role of Bcl-2 apoptotic switch. Our studies here also provide insights into pharmacological manipulation of Bcl-2 family members as cancer therapies. Here the central issue is how to switch the bistable system of Bcl-2 network to on-state and kill tumor cells by manipulating Bcl-2 family proteins. Several BH-3 mimetics have already been designed to sequester anti-apoptotics and trigger apoptosis in tumor cells [38], [39]. We can expect in the future they will be used as a part of normal clinical practice, combining with more precise mathematical modeling which will help to identify specific windows for drug therapies.

In all, through extending the models of Bcl-2 apoptotic switch based on updating experimental findings, we successfully identified two independent positive feedbacks which contribute to the switch mechanism in Bax-activation.

## Supporting Information

Figure S1 Bifurcation diagram of Bax activation as a function of the degradation rate of Bax. Steady states of Activated Bax/Bak (AcBax) are plotted as a function of the degradation rate of Bax.
(0.02 MB PDF)

Figure S2 Influences of the intrinsic noise on the transition of the Bcl-2 apoptotic switch. Possibilities of transition from resting state to on state of Bax activation by changing production rate of Activator under deterministic simulation (solid line) and stochastic simulation (dashed line). For each given value of production rate of Activator, 10000 independent runs are taken for stochastic simulation.
(0.02 MB PDF)

Table S1 Reaction scheme of the Direct Model.
(0.06 MB PDF)

Table S2 Ordinary Differential Equations of the Direct Model.
(0.04 MB PDF)

Table S3 Reaction scheme of the Direct Model I.
(0.06 MB PDF)

Table S4 Ordinary Differential Equations of the Direct Model I.
(0.04 MB PDF)

## References

[1] Saikumar P, Dong Z, Mikailov V, Denton M, Weinberg JM (1999). Apoptosis: Definition, Mechanisms and Relevance to Disease.. Am J Med.

[2] Hengartner MO (2000). The biochemistry of apoptosis.. Nature.

[3] Danial NN, Korsmeyer SJ (2004). Cell death: critical control points.. Cell.

[4] Nair VD, Yuen T, Olanow CW, Sealfon SC (2004). Early single cell bifurcation of pro- and anti-apoptotic states during oxidative stress.. J Biol Chem.

[5] Green DR, Kroemer G (2005). Pharmacological manipulation of cell death: clinical applications in sight?. J Clin Invest.

[6] Loo G, Saelens X, Gurp M, MacFarlane M, Martin SJ (2002). The role of mitochondrial factors in apoptosis: a Russian roulette with more than one bullet.. Cell Death Differ.

[7] Chipuk JE, Bouchier-Hayes L, Green DR (2006). Mitochondrial outer membrane permeabilization during apoptosis: the innocent bystander scenario.. Cell Death Differ.

[8] Kuwana T, Newmeyer DD (2003). Bcl-2-family proteins and the role of mitochondria in apoptosis.. Curr Opin Cell Biol.

[9] Adams JM, Cory S (2007). The Bcl-2 apoptotic switch in cancer development and therapy.. Oncogene.

[10] Galonek HL, Hardwick JM (2006). Upgrading the BCL-2 network.. Nat Cell Biol.

[11] Kim H, Rafiuddin-Shah M, Tu H, Jeffers JR, Zambetti GP (2006). Hierarchical regulation of mitochondrion-dependent apoptosis by BCL-2 subfamilies.. Nat Cell Biol.

[12] Willis SN, Fletcher JI, Kaufmann T, van Delft MF, Chen L (2007). Apoptosis initiated when BH3 ligands engage multiple Bcl-2 homologs, not Bax or Bak.. Science.

[13] Youle RJ (2007). Cellular demolition and the rules of engagement.. Science.

[14] Chen C, Cui J, Zhang W, Shen PP (2007). Robustness analysis identifies the plausible model of Bcl-2 apoptotic switch.. FEBS Lett.

[15] Dlugosz PJ, Billen LP, Annis MG, Zhu W, Zhang Z (2006). Bcl-2 changes conformation to inhibit Bax oligomerization.. EMBO J.

[16] Peng J, Tan CB, Roberts GJ, Nikolaeva O, Zhang Z (2006). tBid Elicits a Conformational Alteration in Membrane-bound Bcl-2 Such That It Inhibits Bax Pore Formation.. J Biol Chem.

[17] Tan C, Dlugosz PJ, Peng J, Zhang Z, Lapolla SM (2006). Auto-activation of the apoptosis protein Bax increases mitochondrial membrane permeability and is inhibited by Bcl-2.. J Biol Chem.

[18] Ruffolo SC, Shore GC (2003). BCL-2 Selectively Interacts with the BID-induced Open Conformer of BAK, Inhibiting BAK Auto-oligomerization.. J Biol Chem.

[19] Willis SN, Adams JM (2005). Life in the balance: how BH3-only proteins induce apoptosis.. Curr Opin Cell Biol.

[20] Li B, Dou QP (2000). Bax degradation by the ubiquitin/proteasome-dependent pathway: involvement in tumor survival and progression.. Proc Natl Acad Sci USA.

[21] Dejean LM, Martinez-Caballero S, Guo L, Hughes C, Teijido O (2005). Oligomeric Bax is a component of the putative cytochrome c release channel MAC, mitochondrial apoptosis-induced channel.. Mol Biol Cell.

[22] Zi Z, Cho K, Sung M, Xia X, Zheng J (2005). In silico identification of the key components and steps in IFN-γ induce JAK-STAT signaling pathway.. FEBS Lett.

[23] Chen C, Cui J, Lu H, Wang R, Zhang S (2007). Modeling of the role of a Bax-activation switch in the mitochondrial apoptosis decision.. Biophys J.

[24] Ferrell JM, Xiong W (2001). Bistability in cell signaling: how to make continuous processes discontinuous, and reversible processes irreversible.. Chaos.

[25] Eißing T, Waldherr S, Allgöwer F, Scheurich P, Bullinger E (2007). Response to Bistability in Apoptosis: Roles of Bax, Bcl-2, and Mitochondrial Permeability Transition Pores.. Biophys J.

[26] Bhalla US (2004). Signaling in small subcellular volumes. II. Stochastic and diffusion effects on synaptic network properties.. Biophys J.

[27] Eißing T, Allgöwer F, Bullinger E (2005). Robustness properties of apoptosis models with respect to parameter variations and intrinsic noise.. IEE Proc-Syst Biol.

[28] Eskes R, Desagher S, Antonsson B, Martinou JC (2000). Bid Induces the Oligomerization and Insertion of Bax into the Outer Mitochondrial Membrane.. Mol Cell Biol.

[29] Letai A, Bassik MC, Walensky LD, Sorcinelli MD, Weiler S (2002). Distinct BH3 domains either sensitize or activate mitochondrial apoptosis, serving as prototype cancer therapeutics.. Cancer Cell.

[30] Gómez-Benito M, Marzo I, Anel A, Naval J (2005). Farnesyltransferase Inhibitor BMS-214662 Induces Apoptosis in Myeloma Cells through PUMA Up-Regulation, Bax and Bak Activation, and Mcl-1 Elimination.. Mol Pharmacol.

[31] Fischer SF, Vier J, Kirschnek S, Klos A, Hess S (2004). Chlamydia Inhibit Host Cell Apoptosis by Degradation of Proapoptotic BH3-only Proteins.. J Exp Med.

[32] Willis SN, Chen L, Dewson G, Wei A, Naik E (2005). Proapoptotic Bak is sequestered by Mcl-1 and Bcl-xL, but not Bcl-2, until displaced by BH3-only proteins.. Genes & Dev.

[33] Bagci EZ, Vodovotz Y, Billiar TR, Ermentrout GB, Bahar I (2005). Bistability in apoptosis: roles of Bax, Bcl-2 and mitochondrial permeability transition pores.. Biophys J.

[34] Legewie S, Bluthgen N, Herzel H (2006). Mathematical modeling identifies inhibitors of apoptosis as mediators of positive feedback and bistability.. PLoS Comput Biol.

[35] Eissing T, Conzelmann H, Gilles ED, Allgöwer F, Bullinger E (2004). Bistability Analyses of a Caspase Activation Model for Receptor-induced Apoptosis.. J Biol Chem.

[36] Bouchier-Hayes L, Lartigue L, Newmeyer DD (2005). Mitochondria: pharmacological manipulation of cell death.. J Clin Invest.

[37] Valentijn AJ, Metcalfe AD, Kott J, Streuli CH, Gilmore AP (2003). Spatial and temporal changes in Bax subcellular localization during anoikis.. J Cell Biol.

[38] Oltersdorf T, Elmore SW, Shoemaker AR, Armstrong RC, Augeri DJ (2005). An inhibitor of Bcl-2 family proteins induces regression of solid tumors.. Nature.

[39] van Delft MF, Wei AH, Mason KD, Vandenberg CJ, Chen L (2006). The BH3 mimetic ABT-737 targets selective Bcl-2 proteins and efficiently induces apoptosis via Bak/Bax if Mcl-1 is neutralized.. Cancer Cell.

[40] Hua F, Cornejo MG, Cardone MH, Stokes CL, Lauffenburger DA (2005). Effects of Bcl-2 levels on Fas signaling-induced Capase-3 activation: molecular genetic tests of computational model predictions.. J Immunol.

