# Supplementary material for: Two Independent Positive Feedbacks and Bistability in the Bcl-2 Apoptotic Switch
Source: PLoS One. 2008 Jan 23;3(1):e1469. doi: 10.1371/journal.pone.0001469 (PMC2194625; doi:10.1371/journal.pone.0001469)

Figure S1 **Bifurcation diagram of Bax activation as a function of the degradation rate of Bax.** Steady states of Activated Bax/Bak (AcBax) are plotted as a function of the degradation rate of Bax.

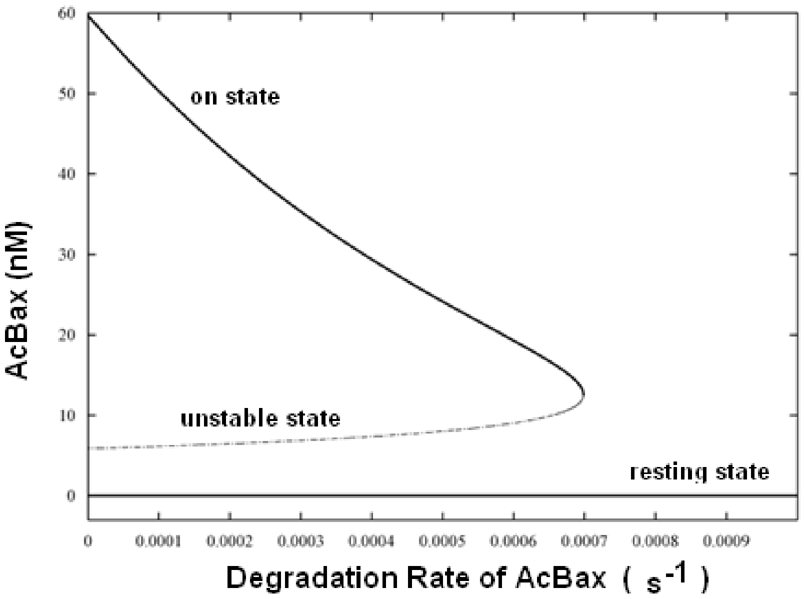

Supplement: Figure S1 — Bifurcation diagram of Bax activation as a function of the degradation rate of Bax. Steady states of Activated Bax/Bak (AcBax) are plotted as a function of the degradation rate of Bax. (0.02 MB PDF) [file pone.0001469.s001.pdf]
