# Supplementary material for: Two Independent Positive Feedbacks and Bistability in the Bcl-2 Apoptotic Switch
Source: PLoS One. 2008 Jan 23;3(1):e1469. doi: 10.1371/journal.pone.0001469 (PMC2194625; doi:10.1371/journal.pone.0001469)

Figure S2 **Influences of the intrinsic noise on the transition of the Bcl-2 apoptotic switch.** Possibilities of transition from resting state to on state of Bax activation by changing production rate of Activator under deterministic simulation (solid line) and stochastic simulation (dashed line). For each given value of production rate of Activator, 10000 independent runs are taken for stochastic simulation.

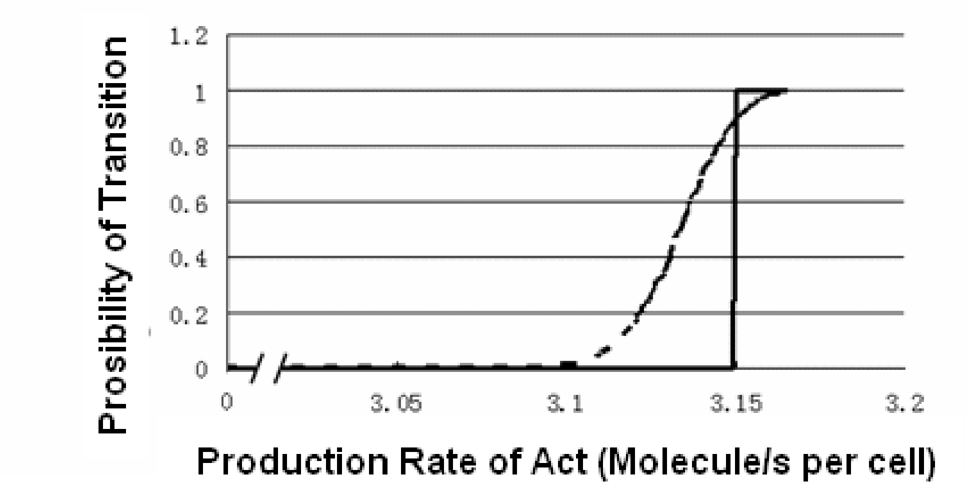

Supplement: Figure S2 — Influences of the intrinsic noise on the transition of the Bcl-2 apoptotic switch. Possibilities of transition from resting state to on state of Bax activation by changing production rate of Activator under deterministic simulation (solid line) and stochastic simulation (dashed line). For each given value of production rate of Activator, 10000 independent runs are taken for stochastic simulation. (0.02 MB PDF) [file pone.0001469.s002.pdf]
