# Supplementary material for: Two Independent Positive Feedbacks and Bistability in the Bcl-2 Apoptotic Switch
Source: PLoS One. 2008 Jan 23;3(1):e1469. doi: 10.1371/journal.pone.0001469 (PMC2194625; doi:10.1371/journal.pone.0001469)
Supplement: Table S1 — Reaction scheme of the Direct Model. (0.06 MB PDF) [file pone.0001469.s003.pdf]

Table S1. Reaction scheme of the Direct Model.[a,b]

Reactions	Description	k+	k-
InBax+Act->AcBax+Act	Act-mediated InBax activation	k1	-
Act+Bcl2<->ActBcl2	Act-Bcl2 dimerization and dissociation	k4	k5
AcBax->InBax	AcBax inactivation	k8	-
Ena+Bcl2<->EnaBcl2	Ena-Bcl2 dimerization and dissociation	k9	k1
Act+EnaBcl2<->ActBcl2+Ena	Displacement between Act and Ena	k12	k1
2AcBax<->MAC	AcBax dimerization and dissociation	k16	k1
InBax<->Φ	InBax degradation and production	p1	u1
AcBax->Φ	AcBax degradation	-	u2
Act<->Φ	Act degradation and production	p2	u3
Bcl2<->Φ	Bcl2 degradation and production	p3	u4
ActBcl2->Φ	ActBcl2 degradation	-	u5
Ena<->Φ	Ena degradation and production	p4	u7
EnaBcl2->Φ	EnaBcl2 degradation	-	u8
MAC->Φ	MAC degradation	-	u9

a. Abbreviations used: InBax (Inactive Bax/Bak), Act (Activator), AcBax (Activated Bax/Bak), Bcl2 (Anti-apoptotics), AcBaxBcl2 (Activated Bax/Bak-Bcl2 dimer), ActBcl2 (Activator-Bcl2 dimer), Ena (Enabler), EnaBcl2 (Enabler-Bcl2 dimer), MAC (Bax/Bak pore), Φ (null).

b. Parameters listed in Table 3 are adopted.
