# Supplementary material for: Two Independent Positive Feedbacks and Bistability in the Bcl-2 Apoptotic Switch
Source: PLoS One. 2008 Jan 23;3(1):e1469. doi: 10.1371/journal.pone.0001469 (PMC2194625; doi:10.1371/journal.pone.0001469)
Supplement: Table S3 — Reaction scheme of the Direct Model I. (0.06 MB PDF) [file pone.0001469.s005.pdf]

Table S3. Reaction scheme of the Direct Model I.[a]

Reactions	Description	k+	k-
InBax+Act->AcBax+Act	Act-mediated InBax activation	k1	-
AcBax+Bcl2<->AcBaxBcl2	AcBax-Bcl2 dimerization and dissociation	k2	k3
Act+Bcl2<->ActBcl2	Act-Bcl2 dimerization and dissociation	k4	k5
AcBax+ActBcl2<->AcBaxBcl2+Act	Displacement between AcBax and Act	k6	k7
AcBax->InBax	AcBax inactivation	k8	-
Ena+Bcl2<->EnaBcl2	Ena-Bcl2 dimerization and dissociation	k9	k1
Act+EnaBcl2<->ActBcl2+Ena	Displacement between Act and Ena	k1	k1
AcBax+EnaBcl2<->AcBaxBcl2+Ena	Displacement between AcBax and Ena	k1	k1
2AcBax<->MAC	AcBax dimerization and dissociation	k1	k1
InBax<->Φ	InBax degradation and production	p1	u1
AcBax->Φ	AcBax degradation	-	u2
Act<->Φ	Act degradation and production	p2	u3
Bcl2<->Φ	Bcl2 degradation and production	p3	u4
ActBcl2->Φ	ActBcl2 degradation	-	u5
AcBaxBcl2->Φ	AcBaxBcl2 degradation	-	u6
Ena<->Φ	Ena degradation and production	p4	u7
EnaBcl2->Φ	EnaBcl2 degradation	-	u8
MAC->Φ	MAC degradation	-	u9

a. Abbreviations and parameters are used as same as in Table S1.
