# Supplementary material for: Two Independent Positive Feedbacks and Bistability in the Bcl-2 Apoptotic Switch
Source: PLoS One. 2008 Jan 23;3(1):e1469. doi: 10.1371/journal.pone.0001469 (PMC2194625; doi:10.1371/journal.pone.0001469)
Supplement: Table S4 — Ordinary Differential Equations of the Direct Model I. (0.04 MB PDF) [file pone.0001469.s006.pdf]

Table S4. Ordinary Differential Equations of the Direct Model I.

$$d[InBax]/dt = J_{InBax} - J_1$$
$$d[AcBax]/dt = J_{AcBax} + J_1 - J_2 - J_4 - J_5 + J_8 - 2 \cdot J_{10}$$
$$d[Bcl2]/dt = J_{Bcl2} - J_2 - J_3 - J_6$$
$$d[Act]/dt = J_{Act} - J_1 - J_3 + J_4 + J_7$$
$$d[ActBcl2]/dt = J_{ActBcl2} + J_3 - J_4 - J_7$$
$$d[AcBaxBcl2]/dt = J_{AcBaxBcl2} + J_2 + J_4 - J_8$$
$$d[Ena]/dt = J_{Ena} - J_6 - J_7 - J_8$$
$$d[EnaBcl2]/dt = J_{EnaBcl2} + J_6 + J_7 + J_8$$
$$d[MAC]/dt = J_{MAC} + J_{10}$$

with

$J_{InBax} = p_1 - u_1 \cdot [InBax]$	$J_1 = k_1 \cdot [InBax] \cdot [Act]$
$J_{AcBax} = -u_2 \cdot [AcBax]$	$J_2 = k_2 \cdot [AcBax] \cdot [Bcl2] - k3 \cdot [AcBaxBcl2]$
$J_{Act} = p_2 - u_3 \cdot [Act]$	$J_3 = k_4 \cdot [Act] \cdot [Bcl2] - k_5 \cdot [ActBcl2]$
$J_{Bcl2} = p_3 - u_4 \cdot [Bcl2]$	$J_4 = k_6 \cdot [AcBax] \cdot [ActBcl2] - k_7 \cdot [AcBaxBcl2] \cdot [Act]$
$J_{ActBcl2} = -u_5 \cdot [ActBcl2]$	$J_5 = k_8 \cdot [AcBax]$
$J_{AcBaxBcl2} = -u_6 \cdot [AcBaxBcl2]$	$J_6 = k_9 \cdot [Ena] \cdot [Bcl2] - k_{10} \cdot [EnaBcl2]$
$J_{Ena} = p_4 - u_7 \cdot [Ena]$	$J_7 = k_{11} \cdot [Ena] \cdot [ActBcl2] - k_{12} \cdot [Act] \cdot [EnaBcl2]$
$J_{EnaBcl2} = -u_8 \cdot [EnaBcl2]$	$J_8 = k_{13} \cdot [Ena] \cdot [AcBaxBcl2] - k_{14} \cdot [AcBax] \cdot [EnaBcl2]$
$J_{MAC} = -u_9 \cdot [MAC]$	$J_{10} = k_{16} \cdot [AcBax]^2 - k_{17} \cdot [MAC]$
